# *Scrophularia peyronii* Post. from Jordan: Chemical Composition of Essential Oil and Phytochemical Profiling of Crude Extracts and Their In Vitro Antioxidant Activity

**DOI:** 10.3390/life13061404

**Published:** 2023-06-16

**Authors:** Yousef Al-Dalahmeh, Sondos Abdullah J. Almahmoud, Nezar Al-Bataineh, Taqwa A. Alghzawi, Abdulrahman G. Alhamzani, Aamal A. Al-Mutairi, Hala I. Al-Jaber, Sultan T. Abu Orabi, Tareq T. Bataineh, Mohammed S. Al-Sheraideh, Mahmoud A. Al-Qudah

**Affiliations:** 1 Department of Basic Pharmaceutical Sciences, Faculty of Pharmacy, Isra University, P.O. Box 33, Amman 1162, Jordan; yousef.dalahmeh@iu.edu.jo; 2 Department of Chemistry, College of Science, Imam Mohammad ibn Saud Islamic University (IMSIU), Riyadh 11623, Saudi Arabia; salmahmoud@imamu.edu.sa (S.A.J.A.); agalhamazani@imamu.edu.sa (A.G.A.); aamutairi@imamu.edu.sa (A.A.A.-M.); 3 College of Pharmacy, Al Ain University of Science and Technology, Abu Dhabi P.O. Box 112612, United Arab Emirates; nezar.albataineh@aau.ac.ae; 4 Department of Chemistry, Faculty of Science, Yarmouk University, P.O. Box 566, Irbid 21163, Jordan; taqwaalghzawi@yahoo.com (T.A.A.); abuorabi@yu.edu.jo (S.T.A.O.); tariq.b@yu.edu.jo (T.T.B.); 5 Department of Chemistry, Faculty of Science, Al-Balqa Applied University, P.O. Box 206, Al-Salt 19117, Jordan; hala.aljaber@bau.edu.jo; 6 Department of Medical Analysis, Faculty of Science, Kurdistan Regional Government, Tishk International University, Erbil 44001, Iraq; 7 Chemistry Department, College of Science, Imam Abdulrahman Bin Faisal University, P.O. Box 383, Dammam 31113, Saudi Arabia; msalsheraideh@iau.edu.sa

**Keywords:** *Scrophularia peyronii*, antioxidant activity, essential oil, *Z*,*Z*-farnesyl acetone

## Abstract

The genus *Scrophularia* is one of the largest genera belonging to the Scrophulariaceae family. Different members of the genus exhibit an interesting, wide spectrum of bioactivities. Accordingly, the current study aimed to investigate, for the first time, the chemical composition of the essential oil of *Scrophularia peyronii* Post. from Jordan. Additionally, extracts obtained from the aerial parts with solvents of different polarities were assayed for their phytochemical constituents and in vitro antioxidant activities. The major constituents detected in the essential oil, as revealed by GC/MS analysis, contained mainly *Z*,*Z*-farnesyl acetone (11.04%), β-elemene (6.36%), *n*-octanal (5.98%), and spathulenol (4.58%). Each of the aqueous methanol (Sp-M) and butanol (Sp-B) extracts contained flavonoids, saponins, anthraquinone, and glycosides. Both extracts were evaluated for their total phenolic content (TPC), total flavonoid content (TFC), and their in vitro antioxidant activity, which were assayed using the DPPH radical scavenging activity and ABTS radical scavenging methods. Additionally, the two extracts were then subjected to LC-ESI-MS/MS for the qualitative determination of their secondary metabolite content, especially in flavonoids and phenolic compounds. The results showed that the Sp-B extract of *S. peyronii* had the highest contents of both phenolic compounds and flavonoids and showed high radical scavenging activity, as determined by the two assay methods, when compared with the Sp-M extract. The LC-ESI-MS/MS analysis resulted in the detection of 21 compounds, including 8 flavonoids, 6 phenolic acids, 6 iridoids, and 2 acids. Although the majority of compounds were detected in both extracts, it was noticed that scropolioside B, 6′-O-cinnamoylharpagide, isoferulic acid, and 6-O-methylcatapol were only detected in the Sp-M fraction.

## 1. Introduction

Oxidative stress, also known as the imbalance between the production of reactive oxygen and nitrogen species (ROS/RNS) and the antioxidant defense, is a significant contributor to several pathophysiological disorders. Oxidative stress is characterized by the inability of endogenous antioxidants to prevent oxidative damage on biological targets. This condition can be brought on by either an increase in the generation of ROS/RNS or a decrease in the antioxidant network [1]. Therefore, considerable attention has been paid to the antioxidant potential of natural products, particularly the most consumable ones.

*Scrophularia* is one of the largest genera of the Scrophulariaceae family. *Scrophularia* is an annual or perennial herb distributed in the Euro-Siberian and Mediterranean regions [2]. In Jordan, there are many species of *Scrophularia*, including *S. peyronii* Post., *S. deserti* Delile., *S. heterophylla* Willd., *S. lucida* L., *S. hierochuntina* Boiss., *S. macrophlla* Boiss., *S. nabataeoeorum* Eig., *S. sphaerocarpa* Boiss., *S. xanthoglossa* Boiss., *S. rubricaulis* Boiss*.,* and *S. xylorrhiza* Boiss. [3]. The use of several *Scrophularia* species in traditional medicine has been described in different cultures of the world. Plants belonging to this genus are utilized for their antioxidant, anti-tumor, anti-cancer, anti-protozoal, and anti-inflammatory effects [4]. Species such as *S. striata* and *S. oxysepala* have been reported for their wound-healing and anti-cancer activity, respectively. The ethanolic extract obtained from the aerial parts of *S. hypericifolia* growing wild in Saudi Arabia has shown hepatoprotective and nephroprotective effects [5,6,7,8,9,10,11,12,13,14,15,16,17,18,19,20,21]. Flavonoids, phenylethanoids, glycoside esters, phenolic acids, iridoids, glycosides, fatty acid derivatives, triterpenes, triterpenoids glycosides, alkaloids, diterpenoids, and essential oils are among the various types of chemical compounds that have been identified in *Scrophularia* species [5,6,7,8,9,10,11,12,13,14,15,16].

The literature survey revealed that *S. peyronii* from Jordan has never been investigated before for its essential oil composition and has not been assayed for its possible antioxidant activity or secondary metabolite contents, especially those of flavonoids and phenolic acids. Therefore, we report in the current study the chemical composition of hydro-distilled essential oil obtained from the aerial parts of *S. peyronii*. Additionally, aqueous methanol and butanol (Sp-M, Sp-B, respectively) extracts of the aerial parts were evaluated for their total phenol content (TPC), total flavonoid content (TFC), and in vitro antioxidant activity. Moreover, the qualitative determination of the phenolic and flavonoid contents of both extracts is performed using LC-ESI-MS/MS.

## 2. Materials and Methods

### 2.1. Instrumentation

UV-vis spectra were measured on a Shimadzu UV-1800 UV/Visible Scanning Spectrophotometer (USA). The essential oil was extracted by the hydro-distillation of the plant’s aerial parts using Clevenger-style equipment. A Varian Chrompack CP-3800 GC/MS instrument was used for the GC/MS (Saturn, The Netherlands). HPLC was utilized to screen molecules of interest in both the positive (M+H) and negative (M-H) electrospray ionization modes using a Bruker Daltonik Impact II ESI-Q-TOF System connected to a Bruker Daltonik Elute UPLC system (Bremen, Germany).

### 2.2. Chemical Reagents

DPPH (2,2-diphenyl-1-picrylhydrazyl, purity N 99%), ascorbic acid (purity 99%), α-tocopherol (purity 99%), ABTS (2,2′-Azino-bis (3-ethylbenzothiazoline-6-sulfonic acid) diammonium salt), Folin and Ciocaltea’s phenol reagent, Na_2_CO_3_, NaOH, anhydrous AlCl_3_, and NaNO_2_ were all products of Sigma-Aldrich (St. Louis, MO 68178, USA).

### 2.3. Plant Material and Fractionations

During the flowering season (April–June; 2019), aerial parts of *S. peyronii* (including stem, leaves, and flowers) were collected from the Al-Kourah region, located in North Jordan (N 32.437537; E 35.690143). The taxonomic identity of the plant was confirmed by the taxonomist Prof. Dr. Jamil N. Lahham (Department of Biology, Faculty of Science, Yarmouk University, Irbid, Jordan). A voucher specimen (No: YU/12/SS/1011) was kept at the Herbarium of Natural Products Research in the chemistry department of the Faculty of Science, Yarmouk University, Irbid, Jordan. The dried plant material was ground into a fine powder (0.50 Kg). Defatting of the dried plant material was performed by soaking it in petroleum ether (40–60 °C, 1 L, ambient temperature, and 10 days). Secondary metabolites were extracted from the defatted plant material by soaking in ethanol (5 times, 7 days each, and 1 L). After the evaporation of ethanol under reduced pressure, the obtained crude extract was partitioned between chloroform and water. Once the chloroform was evaporated, the obtained dried residue was partitioned between 10% aqueous methanol (Sp-M) (7.6 g) and hexane (5.9 g). Polar organic compounds were extracted from the water using n-butanol to obtain the butanol extract (Sp-B) (4.5 g).

### 2.4. Extraction of Essential Oils

In the current investigation, essential oil was extracted from the fresh aerial parts of *S. peyronii*, as described previously in the literature [22,23,24,25,26,27,28,29,30]. Aerial parts of *S. peyronii* (including stem, leaves, and flowers) were chopped into small pieces and then hydro-distillation was carried out in a Clevenger-type apparatus for 4 h. The oil was then dried over anhydrous Na_2_SO_4_ and kept in GC-grade *n*-hexane at 4 °C until the analysis was performed.

### 2.5. Phytochemical Analysis

Each of the Sp-M and Sp-B fractions were subjected to qualitative examination to characterize the major groups of secondary metabolites according to the procedure described by literatures [31,32,33,34].

### 2.6. GC/MS Analysis of Essential Oils

GC/MS analysis of the hydro-distilled essential oil was carried out on a Varian Chrompack CP-3800 instrument (Saturn, The Netherlands). The following was the chromatographic temperature program: 60 °C (isothermal, 1 min), then increased to 246 °C at 3 °C/min, then kept constant at 246 °C (3 min, isothermal); the injector and detector temperatures were 250 and 300 °C, respectively. The carrier gas was helium (0.90 mL/min flow rate). The capillary column used was an HP-5 MS (30 m 0.25 mm i.d., and 0.25 m film thickness). The MS source reached a temperature of nearly 180 °C. A potential of 70 eV was used for sample ionization. A hydrocarbon mixture of *n*-alkanes (C_8_-C_20_) was examined individually by GC/MS using the same HB-5 column under the same chromatographic conditions.

#### Identification of the Chemical Constituents

The identification of the separated essential oil components was accomplished by comparing their calculated retention indices (RIs) with the (C_8_-C_20_) *n*-alkanes values with a column of identical polarity and under the same chromatographic conditions, as well as matching their recorded mass spectra with those listed in the built-in libraries’ spectra (NIST, Gaithersburg, MD, USA and Wiley Co., Hoboken, NJ, USA). The principal components of the extracts were further identified by injecting authentic standard reference compounds under the same chromatographic conditions.

### 2.7. Determination of Antioxidant Activity

The antioxidant activities of the crude extracts and essential oils were determined by the DPPH and ABTS radical-scavenging methods according to the procedure described in the literature [22,23,24,25,26,27,28,29,30]. The scavenging activity was then measured according to the following equation:

(1) $$Scavengingactivity\left(\%\right)=\frac{\left(A_{S}-A_{C}\right)}{A_{C}}\times 100$$

where *A_C_* is the absorbance of the blank and *A_S_* is the absorbance in the presence of the extract.

#### 2.7.1. DPPH Free Radical Scavenging Activity

Briefly, a stock solution from each extract and the obtained essential oil (EO) was prepared (Sp-B, Sp-M, and essential oil; concentration range: 0.005, 0.01, 0.05, 0.1, and 0.5 mg/mL). The absorbance of the solutions was measured using a UV–VIS spectrophotometer at 517 nm against blank samples after allowing them to stand at room temperature in the dark for 30 min. A linear regression approach of plotting the percent of antiradical activity against the concentration of the tested extracts/EO was used to calculate the IC_50_ values, which were defined as the concentration of the substrate that caused a 50% decrease in DPPH activity.

#### 2.7.2. ABTS Radical Scavenging Assay

Briefly, the ABTS^•+^ cation radical solution was prepared by reacting equivalent quantities of 7 mM of ABTS^•+^ and 2.4 mM of potassium persulfate (K_2_S_2_O_8_) solution for 16 h at room temperature in the dark. Prior to use, this solution was diluted with methanol to produce absorbance of 0.75 ± 0.02 at 734 nm. The reaction mixture consisted of 2 mL of ABTS^•+^ solution and 1 mL of each extract (Sp-B, Sp-M)/EO at various concentrations (0.005–0.50 mg/mL). The absorbance of the combination at 734 nm was measured using a UV-Vis spectrophotometer. Each test included a blank and all measurements took a minimum of 5 min to complete. The IC_50_ values were determined by plotting the percent of antiradical activity against the concentration of the tested substances using the linear regression approach.

### 2.8. Statistical Analysis

The presented data were the means ± SD of the results from three independent experiments with similar patterns. Each concentration was tested in triplicate in each of the three independent experiments. Statistical analysis was performed using the one-way ANOVA of the GraphPad Prism 6 software. A *p* < 0.05 value was considered statistically significant.

## 3. Results and Discussion

### 3.1. Chemical Composition of S. peyronii EO

**Figure 1:**
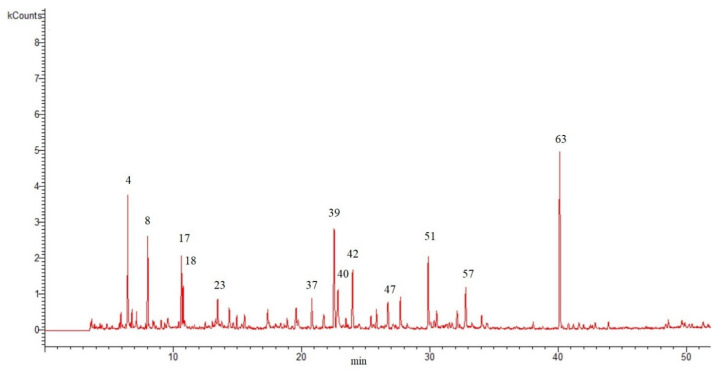
Gas chromatogram for oil extracted from aerial parts of *S. peyronii* obtained from hydro-distillation of the fresh sample (the numbers above the peaks indicate the major chemicals that were identified and are listed by the same numbers in Table 1).

The hydro-distillation of the aerial flowering parts of *S. peyronii* produced a yellowish-colored EO (yielded 0.06% *v*/*w*). The obtained EO was subjected to GC/MS analysis to reveal its chemical constituents. The results are shown in Table 1—the compounds are listed in the table according to their elusion order. Figure 1 shows the GC chromatogram of the analyzed EO.

**Figure 2:**
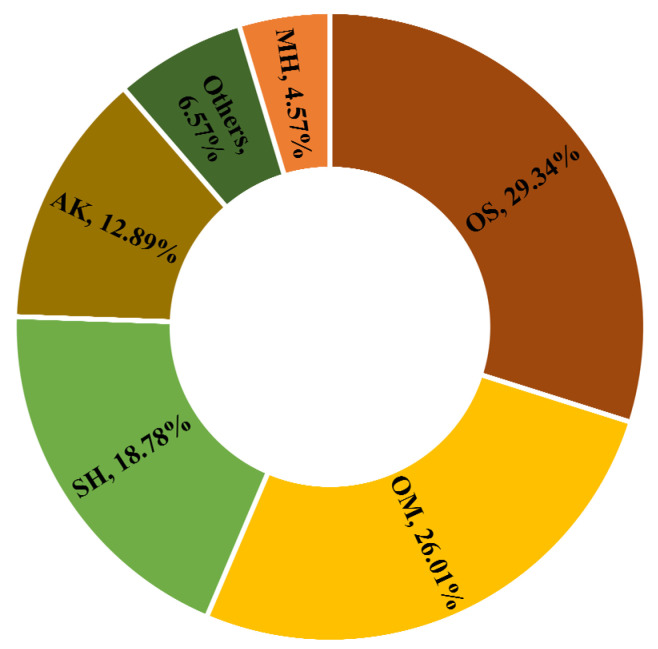
Identified chemical groups from the essential oil of *S. peyronii*; monoterpenes hydrocarbon (MH), oxygenated monoterpenes (OM), sesquiterpenes hydrocarbon (SH), oxygenated sesquiterpenes (OS), aldehyde and ketone (AK), and others.

The results shown in the table grouped the different constituents of the EO into six classes based on their chemical structures that included monoterpene hydrocarbons (MH), oxygenate monoterpenes (OM), sesquiterpene hydrocarbons (SH), oxygenate sesquiterpenes (OS), aldehydes and ketones (AK), and other compounds (Figure 2). The GC/MS analysis resulted in the identification of a total of 66 constituents that represented 98.16% of the total composition (Table 1).

The EO obtained from the aerial flowering parts of *S. peyronii* was dominated by OS that accounted for 29.34% of the total composition. *Z*,*Z*-farnesyl acetone (11.04%) and spathulenol (4.58%) were the main components detected in this class. OM was detected at slightly lower concentrations (26.01%) and was represented mainly by dehydrosabinaketone (3.76%) and citronellyl acetate (2.07%). Other classes found to have appreciable concentration levels in the analyzed EO included SH (18.78%), in addition to carbonyl-containing compounds (AK), which amounted to 12.89% of the total composition.

To the best of our knowledge, this is the first report on the determination of the chemical constituents of the hydro-distilled essential oil extracted from fresh aerial parts of *S. peyronii*. There have been some studies reporting the essential oil composition of other different species of the same genus [35,36,37,38,39]. In these studies, volatile terpenoids, such as humulene, β-caryophyllene, caryophyllene oxide, phytol, linalool, 6α-acetoxymanoyl, and oxide, in addition to non-terpenoidal compounds, such as 1-octen-3-ol, 6,10,14-trimethyl-2-pentadecanone, and pentadecanone, are common in the *Scrophularia* genus [35,36,37,38,39]. In general, it was noticed that oxygenated monoterpenes were the main class of terpenoids detected in the essential oils of plants belonging to the Scrophulariaceae species. The major constituents detected in the essential oils extracted from three *Scrophularia* species growing wild in Iran, including *S. amplexicaulis*, *S. frigida*, and *S. subaphylla*, were eugenol and linalool [37,38,39]. The essential oil extracted from the aerial parts of *Scrophularia deserti* from Vietnam and Iran contained mainly α-pinene [40]. The main constituents of *S. oxysepala* were phytol, methyl benzyl alcohol, dehydrodieugenol, methyl benzaldehyde, and eugenol [35]. Hexahydrofarnesyl acetone, phytol, palmitic acid, β-damascenone, and copaene were the main components of the oil from *S. umbrosa* [41]. The compounds caryophyllene oxide, spathulenol, α-cadinol, and docosane were the main components of the oil from *S. striata* [42].

Our results are consistent with the previous studies conducted on other *Scrophularia* species. However, it was noticed that fatty acids were not detected in our current study. Factors such as those related to the plant’s genotypes, environmental factors, and experimental conditions could justify the differences in the chemical composition of the different plant species of the same genus.

### 3.2. Phytochemical Analysis

The main classes of secondary metabolites in the different fractions obtained from *S. peyronii* extract were determined by chemical methods. This phytochemical analysis revealed unique patterns (Table 2). The Sp-M fraction was found to be rich in alkaloids, flavonoids, saponins, anthraquinone, and glycosides. Fraction Sp-B, on the other hand, contained flavonoids, glycosides, saponins, and anthraquinone. Even though plants synthesize these secondary metabolites for unknown reasons, sometimes as part of their defense system, these different classes of secondary metabolites are important for their pharmacological effects [31,32,33,34].

### 3.3. Phytochemical Profiling of Crude Extracts by Using LC-MS/MS

**Figure 3:**
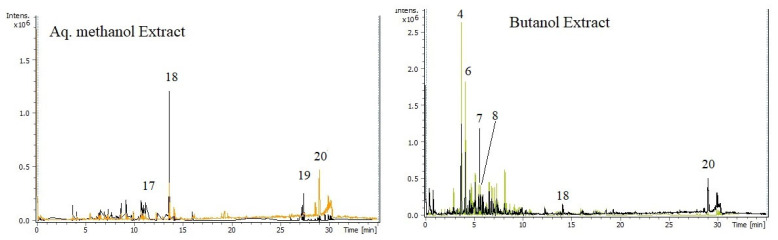
LC-MS chromatogram of Sp-M and Sp-B extracts obtained from *S. peyronii* from Jordan.

In the current investigation, the presence of a selected set of constituents in the Sp-M and Sp-B fractions was determined qualitatively by LC-MS using negative ionization modes. The total ion chromatograms (TICs) for the two fractions are shown in Figure 3. The two extracts were tested for the presence of a selected set of compounds (Table 3), including eight flavonoids, six phenolic acids, six iridoids, and one organic acid. These compounds in general were common to both the genus and the family [4,5,6,7,8,9,10,11,12,13,14,15,16].

In general, the majority of compounds were detected in both investigated fractions (Table 3). It was noticed that, of the six investigated iridoids, three were not detected in the Sp-M fraction. These were scropolioside B, 6′-O-cinnamoylharpagide, and 6-O-methylcatapol. The phenolic compound, isoferulic acid, was also not detected in the Sp-M fraction.

Research on natural product chemistry is driven by the need to isolate and identify new compounds with interesting therapeutic effects. Plants belonging to the Scrophularia genus are well recognized for their use in traditional medicine for the treatment of many ailments, including fever, swelling, and constipation [4]. The presence of a wide spectrum of bioactive secondary metabolites, including glycoside esters, phenylpropanoid glycosides, saponins, and iridoids, is one of these species’ primary distinguishing characteristics. In particular, phenylpropanoids, glycosides, and iridoids are quite common in *Scrophularia* plants and have been found to have apparent therapeutic potential in numerous studies [43]. While phenylpropanoids are well known for their beneficial biological effects, including antioxidant, hepatoprotective, antitumor, and anti-inflammatory properties, *Scrophularia* plants are characterized by the detection of iridoids [43]. Iridoids are quite interesting in terms of their chemical and bioactivity properties. According to information gained from various studies, iridoids isolated from *S. buergeriana* roots were found to have interesting anti-inflammatory, anti-cancer, and antiprotozoal effects [20,44]. E-p-Methoxycinnamic acid, which was isolated from *S. buergeriana*, exhibited anti-amnesic properties and shielded cultured neuronal cells from glutamate-induced neurotoxicity [44]. The isolation of several phenylpropanoid esters, Buergeriside A1, Buergeriside B1, and (E)-p-methoxycinnamic acid from the roots of *S. buergeriana* showed better protection against glutamate-induced neurodegeneration [45,46]. The iridoids scropolioside B and scropolioside D isolated from *S. dentata* showed anti-inflammatory effects [4,45,46].

**Table 3:** The compounds identified in the Sp-M and Sp-B fractions from *S. peyronii* growing wild in Jordan.

No.	Rt (min)	*m*/*z* Meas.	MM Calculated	Name	Structure	Molecular Formula	Fractions		Classification
							Sp-M	Sp-B	
1	0.99	117.0176	118.049	Succinic acid	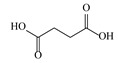	C_4_H_6_O_4_	+	+	Aliphatic acid
2	2.14	375.1257	376.133	3,4-Dihydro-methyl catalpol	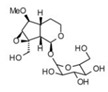	C_16_H_24_O_10_	+	+	Iridoid
3	3.45	625.1359	626.1432	Isorhamnetin-3-O-rutinoside	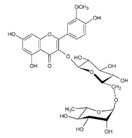	C_28_H_32_O_16_	+	+	Flavonoid
4	3.81	811.2823	812.2896	Scropolioside B	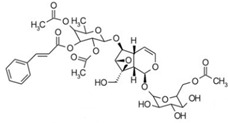	C_41_H_46_O_17_	−	+	Iridoid
5	4.15	179.0325	180.0398	Caffeic acid	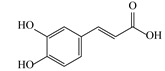	C_9_H_8_O_4_	+	+	Phenolic acid
6	4.49	163.0379	164.0452	p-Coumaric acid	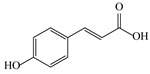	C_9_H_8_O_3_	+	+	Phenolic acid
7	5.41	609.1411	610.1484	Rutin	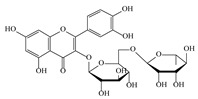	C_27_H_30_O_16_	+	+	Flavonoid
8	5.61	609.1395	610.1467	3-Glu-7-Rha Quercetin	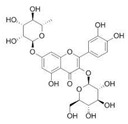	C_27_H_30_O_16_	+	+	Flavonoid
9	5.76	495.203	496.2103	6′-O-cinnamoylharpagide	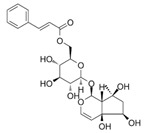	C_24_H_30_O_11_	−	+	Iridoid
10	5.78	175.0377	194.0554	Isoferulic acid	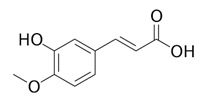	C_10_H_10_O_4_	−	+	Phenolic acid
11	5.78	463.0837	464.0909	Hyperoside	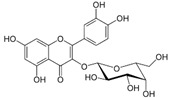	C_21_H_20_O_12_	+	+	Flavonoid
12	6.35	593.1463	594.1535	3-O-Neohesperidoside Kaempferol	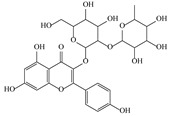	C_27_H_30_O_15_	+	+	Flavonoid
13	6.56	447.0892	448.0965	Kaempferol-3-O-glucoside	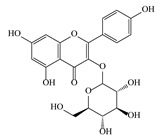	C_21_H_20_O_11_	+	+	Flavonoid
14	6.57	495.3129	496.3202	Nepitrin	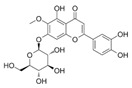	C_22_H_22_O_13_	+	+	Flavonoid
15	7.96	377.0807	756.1759	6-O-Methylcatapol	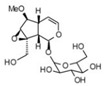	C_16_H_24_O_10_	−	+	Iridoid
16	8.72	147.0427	148.05	Cinnamic acid	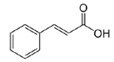	C_9_H_8_O_2_	+	+	Phenolic acid
17	11.09	751.2377	752.2449	Scropolioside A	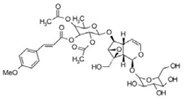	C_35_H_44_O_18_	+	+	Iridoid
18	13.67	752.2377	753.245	Scrovalentinoside	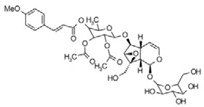	C_35_H_44_O_18_	+	+	Iridoid
19	27.05	478.3855	479.3928	Homoplantaginin	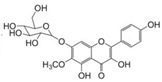	C_22_H_22_O_11_	+	+	Flavonoid
20	29.97	351.2467	352.254	Ningposide C	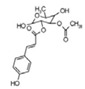	C_17_H_20_O_8_	+	+	Phenolic acid
21	30.07	339.3222	340.3295	3-O-trans-Feruloylrhamnopyranose	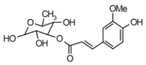	C_16_H_20_O_8_	+	+	Phenolic acid

(+): Detected; (−): not detected; samples were verified against authentic samples isolated in our labs or purchased from Sigma-Aldrich. Rt refers to retention time in minutes, *m/z* refers *to* Mass to charge ratio.

### 3.4. Total Phenolic Content, Total Flavonoid Content, and Antioxidant Activity

The results of the TPC, TFC, and antioxidant activity determined for the two fractions (Sp-M and Sp-B) and the EO obtained from aerial parts of *S*. *peyronii* are shown in Table 4. As could be deduced from the obtained data, Sp-B had the highest TPC and TFC (51.90 ± 1.0 mg/g gallic acid equivalents; 148.54 ± 1.0 mg/g quercetin equivalents) as compared with the other fraction (Sp-M) or EO. The results of the IC_50_ values shown in Table 4 also revealed that, while the DPPH radical scavenging properties of both investigated fractions were comparable, Sp-B had much higher ABTS scavenging activity ((2.5 ± 0.02) × 10^−2^ mg/mL) when compared with the other investigated fraction (Sp-M: (26.0 ± 0.02) × 10^−2^ mg/mL). The observed activity for both fractions could be attributed to the high TPC and TFC of this extract [47,48]. This was also in total agreement with the observed LC-MS/MS results, which revealed the richness of the Sp-B fraction in phenolic acids, flavonoids, and iridoids.

Based on the obtained IC_50_ values, the EO exhibited significant antiradical activity when compared with the employed positive controls (ascorbic acid and α-tocopherol). This could be attributed to the significant content of monoterpene and oxygenated terpenoids, such as limonene, terpineol, (E)-ionone, and citronellol, which are known for their antioxidant properties, all of which were detected in the essential oil obtained from the aerial parts of *S. peyronii* essential oil [49,50]. Additionally, the high content of pulegone and spathulenol, known also for their antioxidant power, may account for the observed high antioxidant activity of the essential oil obtained from *S. peyronii* in our results as compared with the other EOs tested, as indicated in many mint species [51].

## 4. Conclusions

The current study is the first report on the phytochemical screening of *S. peyronii* that included the determination of TPC and TFC, and the evaluation of the antioxidant activity of the two main extracts and the essential oil obtained from the aerial parts of the plant material. Additionally, the essential oil composition and qualitative determination of the selected phenolic compounds and flavonoids were determined by GC/MS and LC-ESI-MS/MS techniques, respectively. The results revealed that the investigated *S. peyronii* extracts had relatively high TPC and TFC, and good antioxidant activity, as determined by the two assay methods (DDPH and ABTS), especially the butanol (Sp-B) extract. The observed antioxidant results were attributed to the presence of numerous phenolic and flavonoid chemicals.

The results of the current study revealed the detection of interesting compounds belonging to the phenolic acid, flavonoid, and iridoid classes. This urges further studies to undertake a detailed phytochemical investigation that is designed to isolate and characterize the active constituents.

## Figures and Tables

**Table 1:** Chemical composition and percentages of essential oil components from aerial parts of *S. peyronii* Post. from Jordan.

No.	Rt (min)	RI _(exp)_	Compound	% Peak Area	Method of Identification
1	3.676	877	(4*Z*)-Hexenol	0.56	MS, RI
2	5.847	954	(2*E*)-Heptenal	0.47	MS, RI
3	5.955	960	Benzaldehyde	0.94	MS, RI
4	6.48	998	*n*-Octanal	5.98	MS, RI
5	6.698	1004	ρ-Mentha-1(7),8-diene	0.47	MS, RI
6	6.819	1007	(2*E*,4*E*)-Heptadienal	0.99	MS, RI, RC
7	7.168	1013	(2*E*)-Hexenyl acetate	0.90	MS, RI
8	8.035	1029	Limonene	4.10	MS, RI
9	8.431	1039	Lavender lactone	0.55	MS, RI, RC
10	8.522	1042	Benzene acetaldehyde	0.52	MS, RI, RC
11	9.086	1059	trans-Decahydro-naphthalene	0.77	MS, RI
12	9.364	1065	Acetophenone	0.52	MS, RI
13	9.579	1081	cis-Vertocitral	0.48	MS, RI
14	9.598	1088	Camphenilone	0.81	MS, RI
15	10.184	1096	Linalool	0.49	MS, RI
16	10.46	1103	2,2-Dimethyl-3,4-Octadienal	0.62	MS, RI
17	10.662	1120	Dehydro-Sabina ketone	3.76	MS, RI
18	10.796	1125	1-Undecyne	2.24	MS, RI, RC
19	10.917	1126	α-Campholenal	1.02	MS, RI
20	12.526	1146	Menthone	0.48	MS, RI
21	13.073	1162	iso-Menthone	0.68	MS, RI
22	13.336	1169	Borneol	0.89	MS, RI
23	13.488	1177	Santalone	2.02	MS, RI
24	13.652	1177	cis-Pinocarveol	0.48	MS, RI
25	13.801	1189	trans-ρ-Mentha-1(7),8-dien-2-ol	0.86	MS, RI
26	14.392	1199	γ-Terpineol	1.57	MS, RI
27	14.699	1217	4-Methylene-Isophorone	0.62	MS, RI
28	14.982	1225	Citronellol	0.92	MS, RI
29	15.589	1237	Pulegone	1.06	MS, RI
30	17.386	1263	(2*E*)-Decenal	1.47	MS, RI
31	17.502	1269	n-Decanol	0.54	MS, RI
32	18.007	1286	5-Undecanol	0.49	MS, RI
33	18.407	1294	Camphorquinone	0.71	MS, RI
34	18.914	1305	iso-Menthyl acetate	0.86	MS, RI, RC
35	19.597	1314	2,3,4-Trimethyl benzaldehyde	1.67	MS, RI
36	19.747	1324	trans-(*E*)-Jasmonol	1.11	MS, RI
37	20.824	1352	Citronellyl acetate	2.07	MS, RI
38	21.755	1375	Linalool isobutanoate	1.29	MS, RI
39	22.565	1393	β-Elemene	6.36	MS, RI
40	22.861	1407	Longifolene	4.30	MS, RI
41	23.487	1408	Dodecanal	0.69	MS, RI
42	24.003	1433	β-Gurjunene	4.16	MS, RI, RC
43	24.549	1439	α-Guaiene	0.48	MS, RI
44	25.428	1459	trans-Prenyl limonene	0.99	MS, RI
45	25.606	1460	allo-Aromadendrene	0.53	MS, RI
46	25.869	1466	(2*E*)-Dodecenal	1.31	MS, RI
47	26.764	1488	(*E*)-β-Ionone	2.06	MS, RI
48	27.158	1502	trans-β-Guaiene	0.61	MS, RI
49	27.714	1513	γ-Cadinene	1.84	MS, RI
50	28.269	1534	trans-Cadina-1,4-diene	0.50	MS, RI
51	29.9	1578	Spathulenol	4.58	MS, RI
52	30.089	1580	n-Hexyl benzoate	0.54	MS, RI
53	30.368	1590	β-Copaen-4-α-ol	0.72	MS, RI
54	30.549	1604	Khusimone	1.52	MS, RI
55	31.757	1623	10-epi-γ-Eudesmol	0.60	MS, RI
56	32.154	1641	allo-Aromadendrene epoxide	1.25	MS, RI
57	32.815	1646	α-Muurolol	3.15	MS, RI
58	33.313	1660	*neo*-Intermedeol	0.66	MS, RI
59	34.06	1679	(*Z*)-Methyl epi-jasmonate	1.07	MS, RI,
60	34.349	1688	Eudesma-4(15),7-dien-1β-ol	0.49	MS, RI
61	34.503	1699	11-αH-Himachal-4-en-1-β-ol	0.48	MS, RI
62	38.085	1792	Drimenone	0.54	MS, RI
63	40.143	1856	*Z*,*Z*-Farnesyl acetone	11.04	MS, RI
64	41.642	1913	(5*E*,9*E*)-Farnesyl acetone	0.60	MS, RI
65	42.907	1942	Callitrisin	0.58	MS, RI
66	43.95	1971	(*Z*)-Methyl-isoprenyl cinnamate	0.53	MS, RI
			Total identified	98.16	

RI refers to the retention index experimentally calculated using C_8_-C_20_ n-alkanes on an HP-5MS capillary column. Rt refers to retention time in minutes. MS refers to identification by mass spectrometry (NIST), and our locally generated libraries were used for all MS comparisons. RC is the identity of the major components and was confirmed by injecting authentic reference compounds into the same chromatography column.

**Table 2:** Secondary metabolite classes detected in the extracts of *S. peyronii* from Jordan.

Groups	Sp-M	Sp-B
Flavonoids	+	+
Glycosides	+	+
Alkaloids	+	−
Tannins	−	−
Saponins	+	+
Anthraquinone	+	+

+: present, −: not present.

**Table 4:** Results of TPC (mg/g gallic acid equivalents), TFC (mg/g quercetin equivalents), and IC_50_ (mg/mL) for the antioxidant activity of the different fractions of *S. peyronii* from Jordan.

Extract	TPC	TFC	IC_50_ (**mg/mL)**	
			DPPH^•^	ABTS
Sp-M	45.44 ± 1.3 *	25.74 ± 2.4	(8.6 ± 0.09) × 10^−2^	(26.0 ± 0.02) × 10^−2^
Sp-B	51.90 ± 1.0	148.54 ± 1.0	(8.0 ± 0.08) × 10^−2^	(2.5 ± 0.02) × 10^−2^
EO	-	-	(4.84 ± 0.12) × 10^−3^	(10.6 ± 0.08) × 10^−3^
Ascorbic acid	-	-	(1.8 ± 0.06) × 10^−3^	(1.9 ± 0.04 ) × 10^−3^
α-tocopherol	-	-	(2.3 ± 0.04) × 10^−3^	(1.8 ± 0.01) × 10^−3^

* Values expressed are means ± S.D. of three parallel measurements. (*p* < 0.05).

## Data Availability

Data are contained within the article.
